# RepeatAnalyzer: a tool for analysing and managing short-sequence repeat data

**DOI:** 10.1186/s12864-016-2686-2

**Published:** 2016-06-03

**Authors:** Helen N. Catanese, Kelly A. Brayton, Assefaw H. Gebremedhin

**Affiliations:** School of Electrical Engineering and Computer Science, Washington State University, Pullman, Washington 99164 USA; Department of Veterinary Microbiology and Pathology, Washington State University, Pullman, Washington 99164 USA

**Keywords:** Short sequence repeat (SSR), Software tool, Knuth-Morris-Pratt algorithm, Visualization, Genetic diversity, Genotyping, *Anaplasma marginale*, Msp1a, RepeatAnalyzer

## Abstract

**Background:**

Short-sequence repeats (SSRs) occur in both prokaryotic and eukaryotic DNA, inter- and intragenically, and may be exact or inexact copies. When heterogeneous SSRs are present in a given locus, we can take advantage of the pattern of different repeats to genotype strains based on the SSRs. Cataloguing and tracking these repeats can be difficult as diverse groups of researchers are involved in the identification of the repeats. Additionally, the task is error-prone when done manually.

**Results:**

We developed RepeatAnalyzer, a new software tool capable of tracking, managing, analysing and cataloguing SSRs and genotypes using *Anaplasma marginale* as a model species. RepeatAnalyzer’s analysis capability includes novel metrics for measuring regional genetic diversity (corresponding to variety and regularity of SSR occurrence). As a part of its visualization capabilities, RepeatAnalyzer produces high quality maps of the geographic distribution of genotypes or SSRs over a region of interest. RepeatAnalyzer’s repeat identification functionality was validated for all SSRs and genotypes reported in 21 publications, using 380 *A. marginale* isolates gathered from the five publications within that list that provided access to their isolates. The tool produced accurate genotyping results in every case. In addition, it uncovered a number of errors in the published literature: 11 cases where SSRs were misreported, 5 cases where two different SSRs had been given the same name, and 16 cases where two or more names had been given to a single SSR. The analysis and visualization functionalities of the tool are demonstrated using several examples.

**Conclusions:**

RepeatAnalyzer is a robust software tool that can be used for storing, managing, and analysing short-sequence repeats for the purpose of strain identification. The tool can be used for any set of SSRs regardless of species. When applied to *A. marginale*, our test case, we show that genotype lengths for a given region follow a normal distribution, while SSR frequencies follow a power-law-like distribution. Further, we find that over 90 % of repeats are 28 to 29 amino acids long, which is in agreement with conventional wisdom. Lastly, our analysis reveals that the most common edit distance is five or six, which is counter-intuitive since we expected that result to be closer to one, resulting from the simplest change from one repeat to another.

**Electronic supplementary material:**

The online version of this article (doi:10.1186/s12864-016-2686-2) contains supplementary material, which is available to authorized users.

## Background

Short-sequence DNA repeats (SSR) are a type of satellite DNA in which a DNA pattern occurs two or more times. These patterns can be homogeneous or heterogeneous, can occur inter- and intragenically, and can be found in both eukaryotes and prokaryotes. While many SSR loci are in noncoding regions of DNA, we focus on those that occur intergenically and thus encode protein repeats as well. These longer intergenic SSRs are of interest as they can be used in genotyping, phylogenetic characterization and analysis of pathogenicity [1]. These loci can be identified for many species in genes encoding diverse functions: for example, SSRs are found in *Haemophilus influenzae yadA*, which encodes an adhesin [2], *Staphylococcus aureus* coagulase, *coa*, which is involved in clotting [3], and *Streptococcus pneumoniae pspA*, a surface protein implicated in pathogenic mechanisms [4, 5].

One species where SSRs have proven useful for genotyping is *Anaplasma marginale*, a bacterial tick-borne pathogen of cattle. Several factors make the development of a vaccine for this pathogen challenging, including the need for a deeper understanding of strain variation and distribution. *A. marginale* Major Surface Protein 1a (Msp1a), a protein with a set of SSRs near the amino terminus, has been extensively used for genotyping strains and cataloguing strain distribution. The *msp1α* repeats are 84–87 bp (28–29 amino acids) in length and vary in sequence and number [6]. To date, over 235 repeat sequences have been identified in the published literature. Cataloguing and tracking these repeats is difficult as diverse groups of researchers are involved in the identification of the repeats. Additionally, the task is error-prone when done manually. For example, there exist cases when the same name has been given to repeats with different sequences (specific instances are included in the Validation section of this manuscript), and conversely, the same repeat has been assigned more than one name (details in the Data Compilation section).

A reliable software tool capable of tracking, managing, analysing and cataloguing SSRs and genotypes can alleviate the aforementioned problems, fuel research and collaboration, and accelerate the path to the discovery of a vaccine. While there exist a variety of tools related to repetitive DNA in the literature, some with a subset of these functionalities, we found that few have sufficient data management capabilities to be used effectively in genotyping and none provide the analysis or visualization functionalities needed to gain insight into geographic genotype distributions. The tools Tandem Repeats Finder [7], scan_for_matches [8] and the ALGGEN software suite [9] can identify user-specified repetitive elements in DNA or protein sequences. However, these tools do not store the search sequences of interest and so they are not suitable to use with a large collection of named repeats. In addition, analysis and visualization functionalities fall outside of the scope of their intended uses. BLAST [10] is a related tool to these with a much more general purpose; it differs from our context, however, in that it enables one to search for a given element in a whole body of genes, rather than a known body of elements in a given gene. Repbase [11] is a database that stores repetitive DNA elements for a number of species for use in other tools. The tools that use the Repbase data, such as CENSOR [12] and RepeatMasker [13], are largely focused around removing repetitive elements rather than tracking or analysing them. Additionally, Repbase is limited to data from eukaryotes, which in turn limits the tools built around it. PSSRdb [14] is a database that stores repetitive gene data for many species of prokaryotes, including *A. marginale* and *S. pneumoniae*, but focuses on shorter repeating elements (1–6 bp) called simple sequence repeats. There is another class of related tools, with functionality not directly related to genotyping, that identify general repeating elements in DNA or protein sequences in a variety of ways. Examples of tools that fall into this category include [7, 15–24]. While there are no tools specifically related to genotypic and geographic repeat analysis, we note that RepeatFinder [21] has some interesting analysis capabilities involving substructures of the repeating elements it identifies. Finally, we note that repeat finding in a broader sense is an extensively studied topic in bioinformatics. Sharma et al [25] provide a survey of tools used for mining microsatellites in eukaryotic genomes. Merkel and Gemmel [26] give a review of software tools for detecting short tandem repeats and a practical guide (aimed at biologists) on how to use the tools in an informed manner. Lim et al [27] provide a more recent review of tandem repeat search tools with a focus on algorithmic performance.

We developed RepeatAnalyzer, a new software tool designed to track, manage, analyse and catalogue SSRs and genotypes. The idea for RepeatAnalyzer originated from a very simple program for repeat identification developed by Carter Hoffman [28], and branched out to add many new features and analysis capabilities. Using a summary of repeat and strain data collected from the literature, RepeatAnalyzer currently maintains a list of all known *A. marginale* repeats and strains, the locations where these have been reported, and the original sources of those reports. When given the DNA or amino acid sequence of a gene/protein of interest, RepeatAnalyzer can determine which repeats the sequence contains and whether the genotype has been previously reported. RepeatAnalyzer also provides a variety of analysis capabilities, including: genetic diversity analysis for a specified geographical region; genotype analysis for a specified SSR, strain or location; and visualization of search results on geographic maps. While we developed RepeatAnalyzer using *A. marginale* as the model organism, the tool can also be used in the study of other bacteria with SSR loci.

## Implementation

**Fig. 1:**
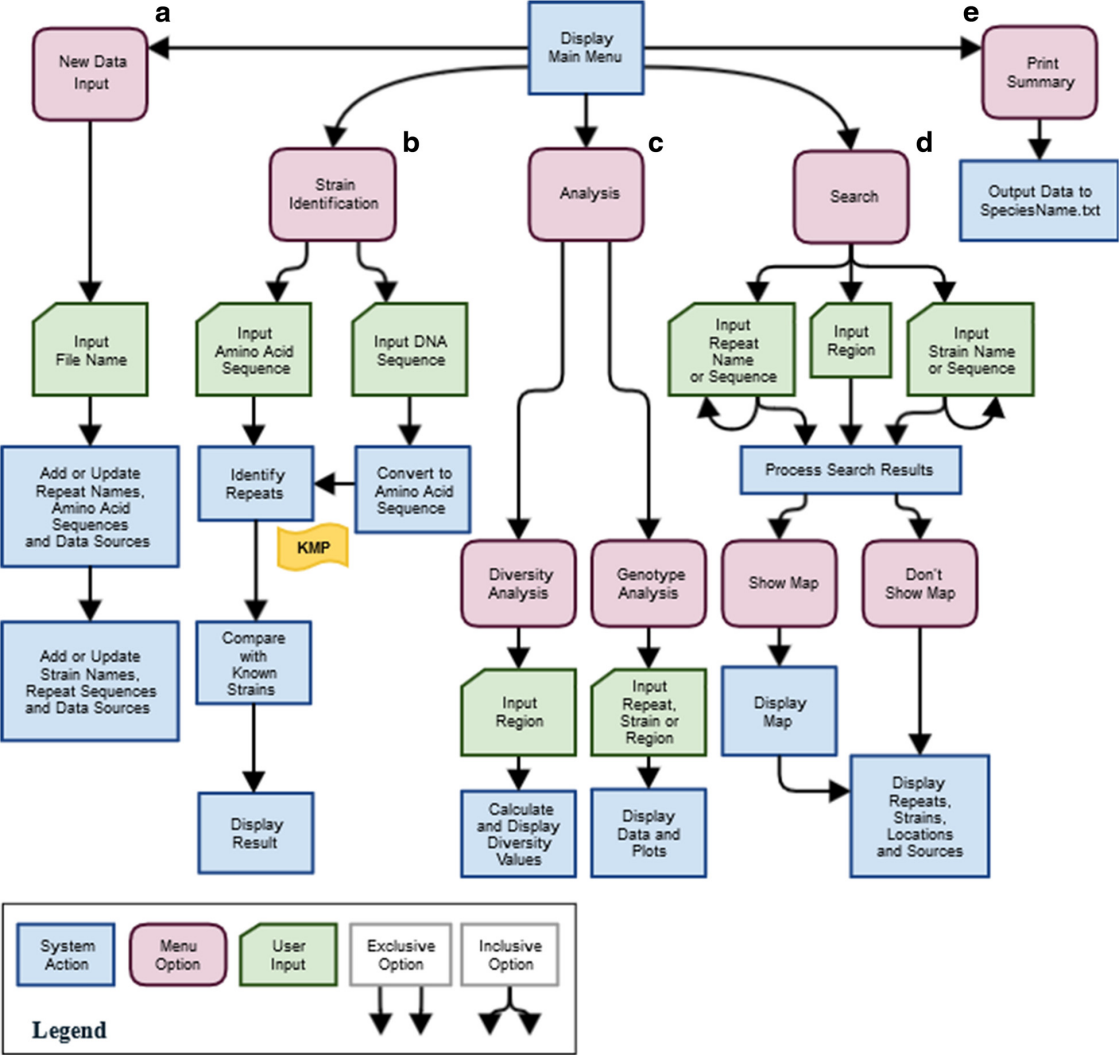
A flowchart of RepeatAnalyzer’s functionalities. Each main branch (**a**-**e**) represents a type of functionality. Following each branch shows the program flow for each option

RepeatAnalyzer is a command line software tool, written in Python, for storing, managing, identifying and analysing a catalogue of SSRs for a gene locus of interest. These functionalities can be used in genotyping, phylogenetic characterization or pathogenic analysis. Figure 1 provides an overview of the central functionalities of RepeatAnalyzer.

### RepeatAnalyzer functionalities

A) *Input Newly Discovered SSRs and Genotypes.* RepeatAnalyzer can add new genotypes and SSRs to its dataset from a plain text file specified by a user. The file is required to be in a simple but specific format (details are provided in the user manual). If data in the file already exist in the system, RepeatAnalyzer avoids adding the data again. Further, the program carries out a variety of checks to ensure consistency of data added to the dataset. If an occurrence of a genotype is found at a new location, that location is recorded. If a genotype or SSR with a known sequence is given a new name, that name is added to a list of current names for that genotype or repeat. If a repeat name that is in use is listed with a new sequence, the user is prompted to overwrite the old sequence, keep it in lieu of the new one or give it a new (unused) name. This is done to maintain the uniqueness of repeat names, which is necessary as the repeats present in a genotype are referenced by name only. The data input functionality of RepeatAnalyzer is shown in branch A of Fig. 1.
B) *Strain Identification.* Given the sequence (either in DNA or amino acid form) of a known SSR locus for an organism from a species of interest, RepeatAnalyzer finds the maximal set of non-overlapping known repeats in that sequence and the name of the genotype, if any is stored. It also gives the locations where the genotype has been found previously and the sources of this information. The SSRs used for this functionality must have been input as described in section A. As mentioned in the Data Compilation section, the input file for *A. marginale* will be maintained along with the software, and files for other species may be kept similarly, if there is sufficient interest. However for any other species, the input file will need to be compiled by the user, as specified in the user manual.

**Fig. 2:**
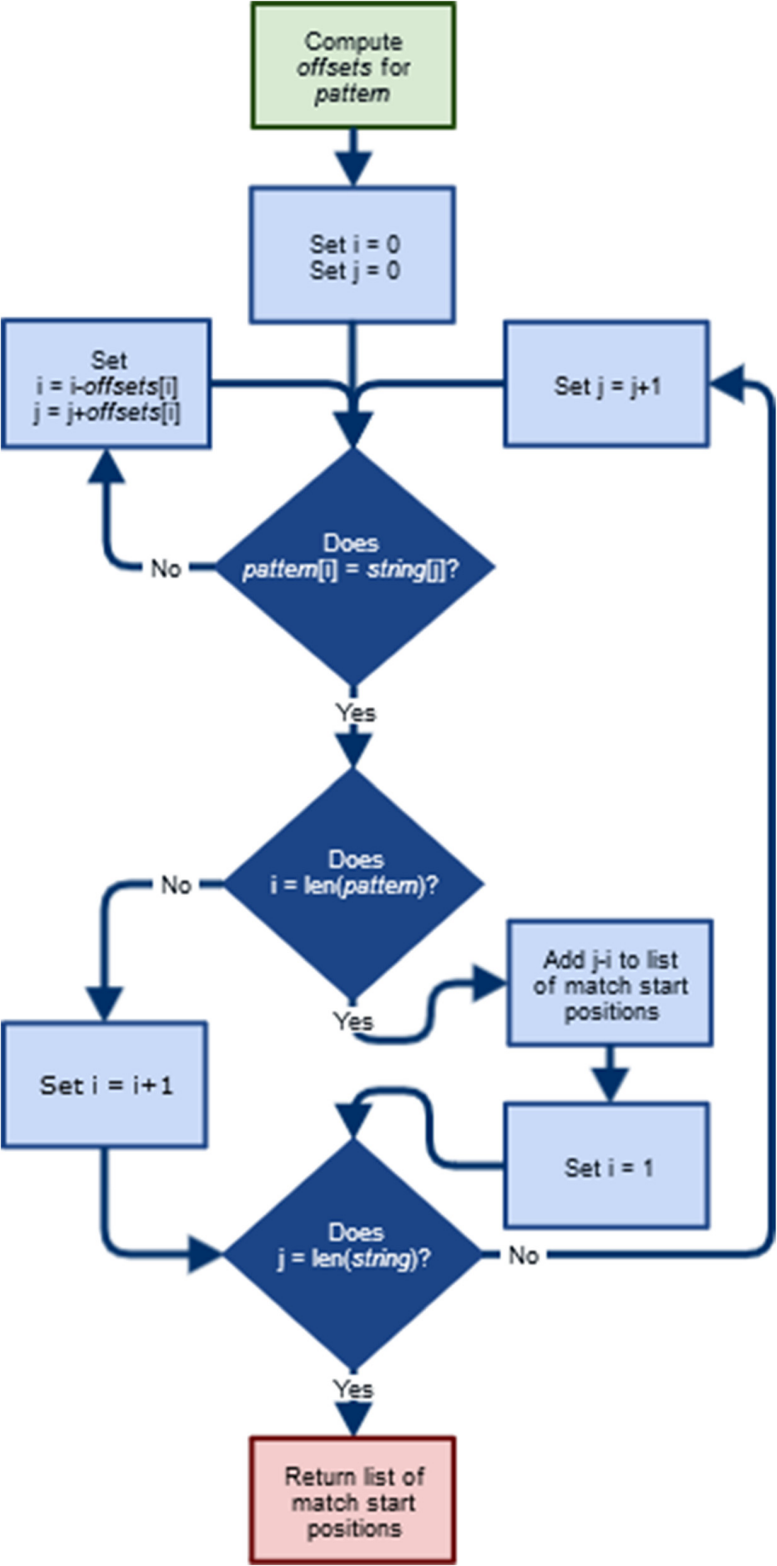
A flowchart of the Knuth-Morris-Pratt string searching algorithm. KMP is a computationally efficient algorithm for finding short text patterns in a long string of characters. *offsets* is a list of numbers. The length of *offsets* is the number of characters in *pattern*. Each entry in *offsets* corresponds to the distance counters i and j are adjusted when a mismatch occurs. len(*item*) denotes the length of *item*

The strain identification functionality is represented in branch B of Fig. 1. Upon selecting the appropriate menu item, the user can enter either the DNA or amino acid sequence for the gene of interest. Once the user has selected the appropriate option for the data entered, the program converts the input to amino acid form (if it is provided in DNA form), and then runs an implementation of the Knuth-Morris-Pratt (KMP) string searching algorithm [28] to search for all known repeats in the string (a schematic description of the KMP algorithm is provided in Fig. 2). Once this is completed, the program removes repeats that are substrings of other found repeats from the results before they are output. In the search for repeats, we chose the KMP algorithm because it is fast in practice (linear time) and has guaranteed worst-case behaviour compared to theoretically faster algorithms that do not offer the same reliability such as Boyer-Moore [29]. Suffix trees [30], which facilitate more efficient search procedures, are not suitable in our context because the search body is input at runtime (the time to build these structures, which is on the same order as running KMP itself, would counteract their search efficiency while consuming additional memory).

C) *Analysis.* RepeatAnalyzer provides two basic analysis functionalities for the repeat data it stores, which we have called diversity analysis and genotype analysis.
  i) *Diversity analysis* involves calculation of genetic diversity scores for a geographic region. A region can be defined as broadly as a country or as narrowly as a particular province or county. The genetic diversity is calculated in several different ways that we group into two categories. The first category of metrics (quantity-centric) measures the percentage of unique repeats in a region, while the second (distribution-centric) measures the regularity with which the repeats are distributed. The metric GD2, which was introduced in [31], is a variant of the quantity-centric category. It is simply the number of unique SSRs present in a region divided by the number of strains identified. We introduce a slightly modified variant of GD2, named GD2b, that can be calculated with only genotype data, as this is what RepeatAnalyzer stores. Specifically, GD2b is defined as the number of unique SSRs divided by the number of genotypes. In addition, we developed a new quantity-centric measure of diversity (called GDM1), and a new distribution-centric diversity measure (called GDM2). GDM1, like GD2 and GD2b, measures the amount of unique repeats in a region, but unlike GD2 and GD2b, is unaffected by the length of genotypes in the region. (Details on this are included in the Discussion). GDM2 measures how uniformly the repeat occurrences in a region are distributed. GDM1 and GDM2 each come in two variants, *local* and *global*, depending on whether the metric is calculated as an average of the values for each genotype or over the entire region, respectively. The mathematical definitions of these measures are summarized in Table 1.

**Table 1:** Metrics used to calculate genetic diversity

Metric Name	Formula	Significance
GD2	$\left(\frac{Total\ \#\ Unique\ SSRs}{\# Strains}\right)\times 100$	Defined in [31]
GD2b	$\frac{Total\ \#\ Unique\ SSRs}{\#\ Genotypes}$	Modified version of GD2, calculable in RepeatAnalyzer
GDM1-Local	$Avg\left(\frac{\#\ Unique\ SSRs\ in\ Genotype\ i}{Length\left( Genotype\ i\right)}\right)$	Ratio of unique repeats in each strain in the region
GDM1-Global	$\frac{Total\ \#\ Unique\ SSRs}{Total\ Length\left( All\ Genotypes\right)}$	Ratio of unique repeats across the region as a whole
GDM2-Local	$Avg\left( Deviation\left(\frac{Frequency\left(SSR\ i\ in\ Genotype\ j\right)}{Length\left( Genotype\ j\right)}\right)\right)$	Variation in how often repeats occur within strains in the region
GDM2-Global	$Deviation\left(\frac{Frequency\left(SSR\ i\right)}{Total\ Length\left( All\ Genotypes\right)}\right)$	Variation in how often repeats occur in the region as a whole

Length(Genotype) = the number of SSRs in that genotype
    In addition to these numeric genetic diversity measures, RepeatAnalyzer calculates the frequency of each SSR in the region (by the number of genotypes in which it occurs) and lists the set of SSRs that are unique to the given region. Plots can (optionally) be produced of the distribution of repeat frequencies, repeat lengths or genotype lengths.
  ii) *Genotype analysis*, the second type of analysis RepeatAnalyzer provides, is a detailed summary of the compiled information about a single SSR, genotype or location. As an example, the compiled data for *A. marginale* SSRs in Mexico would include all genotypes found in Mexico, all SSRs in those genotypes, and all papers referencing these genotypes in Mexico or elsewhere in the world. The analysis for a particular genotype includes: a list of all the repeats the genotype contains, the edit distances (the number of amino acid differences between two repeats) between the repeats in the genotype and the mean edit distance; a list of all locations where the genotype has been reported; and a list of all papers reporting a strain with the genotype. For an SSR of interest, RepeatAnalyzer prints a summary of all locations where the repeat has been found, the genotypes in which the repeat is found and the sources in which the findings were reported as well as any SSRs within a given edit distance from that repeat’s sequence and any other SSRs of which it is a subset.
    The analysis functionality of RepeatAnalyzer is summarized in the C branch of Fig. 1.

D) *Search.* While similar to the genotype analysis functionality, search returns a more succinct summary of information on a large number of repeats and/or strains at once, and it maps these results geographically. For a genotype of interest, a search could be performed on the genotype and/or a repeat present in the genotype. The search produces a map of every location where the genotype has been found as well as every instance of its composite repeats. Alternately, a set of similar genotypes could be searched to show where each of them occurs, or all genotypes of a region could be mapped. Examples of this functionality are shown in the Results section.

The search functionality is depicted in the D branch in Fig. 1. The input here can include one or more strains, one or more repeats, and a region. It is also possible to look for all strains or all repeats in a given location or all instances of a given strain or repeats across all geographic regions. The program processes this query and the user may choose to include a map plot of their search as well. The plot shows the locations of all the searched strains and repeats over a world map that can be zoomed to any region of interest. These maps can be saved in most popular image formats (for high resolution images the PDF or SVG formats are recommended) and the data printout can be copy-pasted into a word processor or spreadsheet.

E) *Summarise All Current Data.* RepeatAnalyzer can also print a summary of all the repeats and strains it is currently storing, along with all the locations where these have been recorded and the sources. The printout is stored in a text file in the same folder as the executable file. If a strain or repeat has multiple names, the names are separated by semicolons. This simple functionality is shown in the E branch of Fig. 1.

### Data compilation

To build, test and verify RepeatAnalyzer, we used *A. marginale* SSRs and genotypes we compiled by mining the literature. Specifically, we conducted a review of the literature examining papers that reported new SSRs or repeat sequences for genotyping. A total of 25 papers were reviewed and 21 were found to have genotype and/or repeat data [6, 31–50].

We found 16 cases in the literature [32, 35–37, 40, 45, 47, 50] where the same nucleic acid sequence was assigned a new name independently. When this occurred, we counted it as a single repeat with multiple names. Similarly, we counted unique genotypes only where a distinct sequence of repeats existed and thus the total number of unique genotypes we identified is significantly lower than the total number of strains reported. A table of all unique repeat sequences and all their published names is available in Additional file 1. We designed our software to account for the potential problem of multiple naming of SSRs by alerting users to existing SSRs with a given name before they are input into the program and allowing them to be renamed.

## Results

In this section we demonstrate various functionalities of RepeatAnalyzer using (a) the *A. marginale msp1α* data we collected to validate RepeatAnalyzer’s functionalities and (b) some preliminary *S. pneumoniae* PspA SSRs and genotypes we identified. First, we show RepeatAnalyzer’s correctness on a large number of inputs and outline several types of errors we found in the literature in the process that the storage and management features of RepeatAnalyzer could prevent in the future. Next, we show several examples that illustrate the mapping and analytic capabilities of RepeatAnalyzer. Finally, we identify some preliminary PspA SSRs and genotypes and compare our genotyping strategy to one used in the *S. pneumoniae* literature.

### Validation

In order to validate RepeatAnalyzer’s identification functionality we downloaded 380 published *A. marginale msp1α* sequences [31, 32, 36, 39, 42] from GenBank [51]. Using RepeatAnalyzer we compared the sequence of the repeats in GenBank against the sequence the isolates were reported as having in the corresponding publication. Of the 380 sequences examined, the results returned by RepeatAnalyzer corresponded with the literature in 369 cases. For the remaining 11 cases, manual curation revealed that the information reported by RepeatAnalyzer was correct in all of the cases, assuming the GenBank data is correct. Of the 11 erroneous cases, only five presented unique errors. In addition to these, RepeatAnalyzer had previously corrected five instances of a single name being assigned to two different repeats, which would have otherwise resulted in mismatches. For these 10 distinct errors, by comparing the published repeat sequence to the sequence in GenBank, we were able to classify the errors into two broad categories as i) mistaken repeat sequence and ii) mistaken repeat name.

i) *Mistaken Repeat Sequence.* This error occurs when the published sequence of an SSR is different from the actual SSR sequence present in GenBank. Often the difference between the two sequences is small. This type of mistake is easy to make when dealing with a large number of sequences by hand, and it can lead to major confusion when a genotype is effectively mischaracterized as consisting of the wrong series of repeats. RepeatAnalyzer can prevent such errors by allowing researchers to simply enter the sequence of interest and have a known repeat returned with its name(s), avoiding the need to tediously compare each amino acid one by one. In most cases, we found that the mistaken repeat sequence is actually a new SSR. In such cases, we renamed the sequences by adding a “-2” to the end of the reported name. Instances of the mistaken repeat sequence we found are summarized in the top part of Table 2.

**Table 2:**
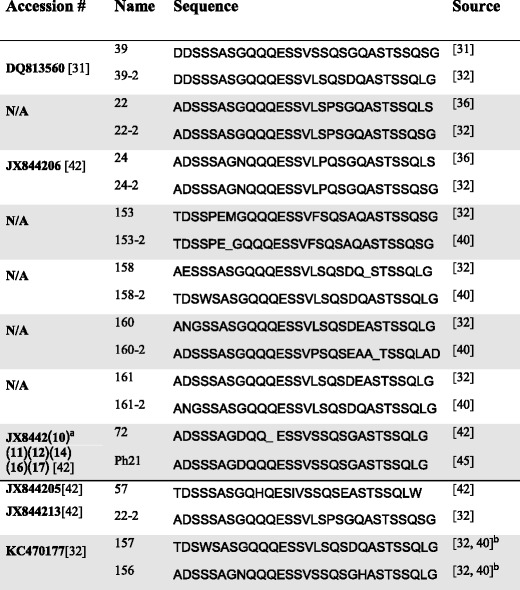
Mistaken repeat sequence and mistaken repeat name found in the *A. marginale* literature

^a^Each value in parentheses is the last two digits of an accession code where the first digits precede the parenthesized values

^b^This repeat was listed as co-reported in the indicated papers
ii) *Mistaken Repeat Name.* This error occurs when the SSR is reported as corresponding to a previously published SSR, but actually corresponds to a different published SSR. While it is relatively easy to avoid, a mistake like this can be nearly impossible to notice without reanalysing the original sample. In the sequences we examined, we found fewer cases of mistaken repeat names than of mistaken repeat sequences. RepeatAnalyzer could also prevent mistaken repeat name errors, since the set of SSRs in a sequence is returned by their names, removing the need for any manual checking. Instances of the mistaken repeat name we found are summarized in the bottom part of Table 2.

### Examples using *A. marginale* data

Because there is already a large collection of *A. marginale* SSRs (over 235) and genotypes (over 350) available, we frame the following examples around the *A. marginale* data we collected. But the same analysis can easily be applied to SSRs and genotypes for any prokaryote that has distinct SSRs. In a subsequent section we will give an example of another species for which SSR genotyping could provide beneficial insights and for which RepeatAnalyzer’s functionalities work seamlessly.

#### Regional diversity analysis

RepeatAnalyzer has several ways to characterize the diversity within a given geographical region. First, it can easily compute the frequency, within known genotypes, of each SSR that occurs in a region. Second, it can show the list of SSRs unique to a given region (by unique we mean that they have not been reported in an isolate from any other area). RepeatAnalyzer can also produce plots of distributions of SSR frequency, SSR length and genotype length. Finally, it can produce a variety of diversity scores that characterize SSR frequency more succinctly.

As an example, we include the diversity scores for two provinces of Mexico, Nayarit and Jalisco, and for Kansas, USA in Table 3. The table also includes worldwide scores for comparison. Based on GDM1-Local scores, we can see that Kansas has relatively few unique SSRs per genotype, compared to both the Mexican provinces and the world, while Jalisco has relatively diverse SSRs within its genotypes. Similarly, Kansas has low GDM1-Global value, meaning low SSR diversity in general, while Jalisco has a higher value for the same metric. For both GDM2 metrics, the world values are the lowest, which is expected as these values contain all known repeats, most of which occur only a few times. The Kansas scores, both in local and global terms, are relatively high meaning that, both within each genotype and in general, genotypes contain a more uneven mix of SSRs (i.e. many of one repeat and few of several others). In contrast, the two Mexican provinces have lower GDM2 scores, indicating that the repeats are more evenly distributed. Both Mexican provinces were found to have repeats unique to them: for Jalisco LJ2 is unique and for Nayarit EV1, EV5, EV9, and EV10 are unique. Kansas has fewer SSRs in general, and none are unique.

**Table 3:** Diversity scores for Nayarit and Jalisco, Mexico, Kansas and world data

Metric	Nayarit	Jalisco	Kansas	World
GD2b	70	145	56	77
GDM1-Local^a^	0.692	0.814	0.395	0.739
GDM1-Global^a^	0.148	0.345	0.114	0.177
GDM2-Local^a^	0.095	0.074	0.103	0.092
GDM2-Global^a^	0.027	0.028	0.196	0.010

^a^These values are rounded, however as they are constructed from counts, rather than measurements, they do not have a strict number of significant digits. Rather, for the GDM scores, we have chosen to display numbers rounded to three significant digits to make it easier to read and compare them. For the GD2b score we decided to round to whole numbers as the magnitudes are of a different scale

**Fig. 3:**
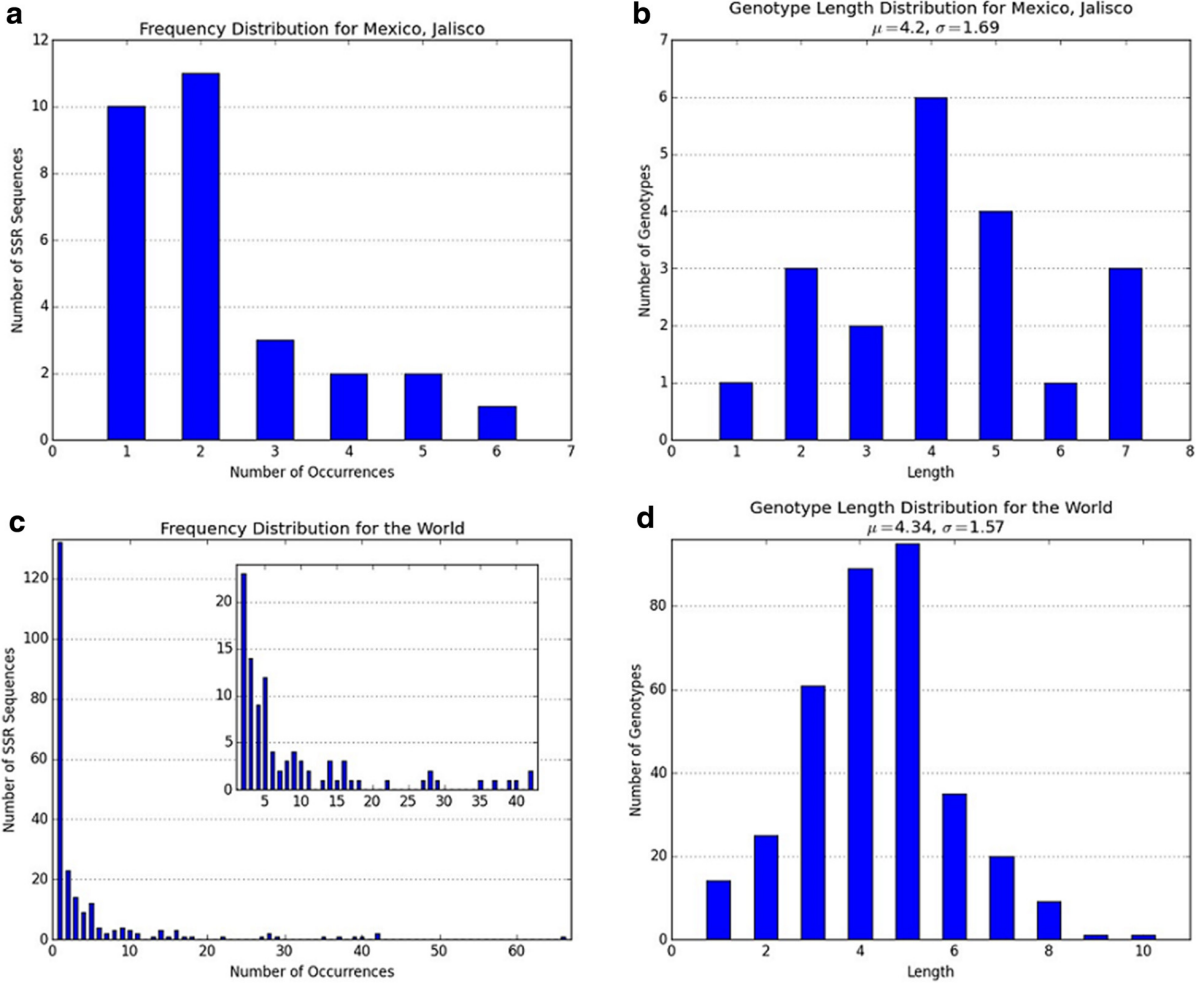
Repeat frequency and genotype length distributions. The four plots in the figure are histograms produced by RepeatAnalyzer for Jalisco, Mexico and whole world data. Plots **a** and **c** show the distribution of SSR frequencies by the number of genotypes in which they occur in the region. Plots **b** and **d** show distributions of genotype lengths. The Inset in figure **c** is zoomed in to show its middle segment in finer detail; RepeatAnalyzer automatically generates this type of inset when outlier values would make the indices on the table difficult to interpret

The plots RepeatAnalyzer produces for the SSR frequency and genotype length distributions for Jalisco and the world data are included in Fig. 3. We found that generally genotype lengths are normally distributed around a mean for any given region, though the mean varies, while frequencies follow a power-law resembling distribution. Further, our analysis of SSR frequency distribution plots for various regions (data not shown) showed that over 90 % of repeats are 28 to 29 amino acids long, in agreement with what is commonly believed by researchers. In addition, we found that repeats of length 28 are approximately 0.35 times as likely as repeats of length 29 (with a standard deviation of 0.15). This is consistent with the global average ratio, where repeats of length 28 occur 0.34 times as often as repeats of length 29.

#### Genotype analysis

In addition to characterizing the level of diversity in a geographical region, RepeatAnalyzer can also be used to compare and analyse individual entities (either genotypes or SSRs). As an example, we discuss results we obtained for genotype α β β Γ, originally reported in Mexico. The analysis revealed that the genotype has been reported in Minas Gerais, Brazil and various locations around Mexico and that the average edit distance (found to be 8) between its SSR sequences is fairly high. For each repeat in the genotype, the analysis also showed every reported genotype containing that repeat and where it was found. Many of the locations where these repeats appear are similar, including a variety of provinces around Mexico, Brazil and Venezuela, in addition to Taiwan. However β and Γ are also reported in the Philippines and Γ is reported in Italy.

Further, when the investigator enters the desired edit distance, genotype analysis can identify repeats with one or more amino acid substitutions, in addition to the known SSR with the furthest edit distance. A summary of the results of such an analysis for the repeats in genotype α β β Γ are included in Table 4 together with results for other common SSRs for comparison. In this example, we searched with a maximum edit distance of three. Interestingly, α is found to have only two recorded repeats within edit distance 3, with repeat 108 at edit distance 1 and repeat Ph1 at edit distance 2, while β has more SSRs at each edit distance. Both α and β have significantly fewer similar SSRs (with an edit distance less than 3) than the other repeats analysed, including E and 27 which were selected because they have been reported in a similar number of regions as α and β. When examining the relationship of the repeats to each other, the simplest change would be a single amino acid change from the repeat being examined. Ergo, one might expect a given repeat to have the most common edit distance as 1 when comparing to other repeats. However, analysis of edit distances shows that the most common edit distance for the 235 *A. marginale* repeat data set is 5 or 6, with a slightly skewed normal distribution around this point (the distribution is skewed in that it has a longer tail to the right of the highest point). We posit that this is result of long evolutionary time, where multiple amino acid substitutions have accumulated and been selected for.

**Table 4:** Edit distance analysis results for some common repeats

	# SSRs reported at edit distance (ED):					
SSR	1	2	3	Max	Max ED	Reported in
α	1 (108)^a^	1 (Ph1)	0	1 (135)	16	Mexico, Brazil, Argentina, Taiwan, Venezuela
β	6	6	10	2 (99, EV6)	16	Mexico, Brazil, Argentina, Taiwan, Venezuela, Philippines
Γ	10	16	44	4 (99, 134, 135, EV6)	12	Italy, Mexico, Brazil, Argentina, Taiwan, Venezuela, Philippines
E	10	20	31	1 (135)	13	United States, Puerto Rico, Israel, Venezuela, Mexico
27	15	46	51	4 (133, 135, EV6, EV11)	12	Argentina, South Africa, Brazil, Philippines, Mexico, Venezuela,
M	11	34	63	1 (135)	13	United States, Brazil, Italy, Argentina, Israel, Mexico, Philippines, Venezuela, South Africa, Taiwan

^a^Parentheses contain the name of the referenced repeat

#### Geographic distribution visualization

**Fig. 4:**
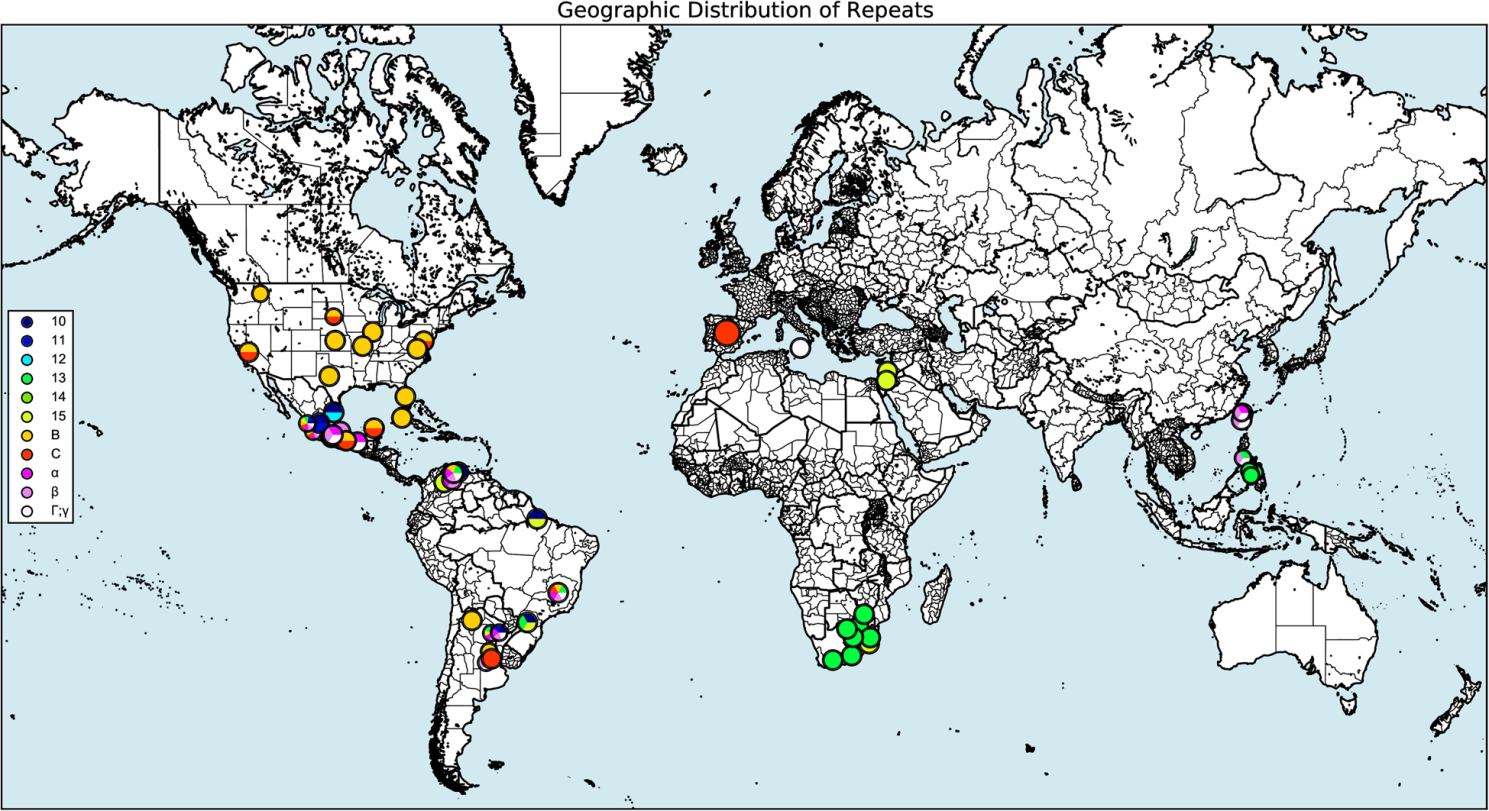
Geographic visualization of repeats. The figure shows the output of the query: Repeats: 10; 11; 12; 13; 14; 15; B; C; α; β; Γ, Strains: None, Location: Any, Scale: 1. A version of this same map zoomed in to show Venezuela in detail is available in Additional file 2. The size of a circle indicates the scope of the region it denotes. This is necessary because while a location for a genotype must include a country, it may also (optionally) include a province and/or county. In these cases, the larger the circle is, the broader the scope; country only markers have the largest circles, while markers for a specific county are the smallest

**Fig. 5:**
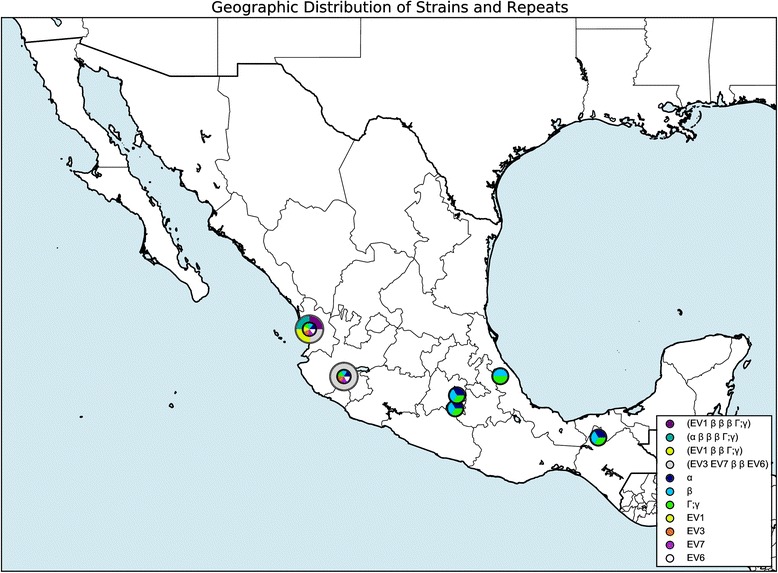
Geographic visualization of repeats from Nayarit, Mexico. The figure shows the output of the query: Repeats: α; β; Γ; EV1; EV3; EV7; EV6, Strains: EV1 β β β Γ; α β β β Γ; EV1 β β Γ; EV3 EV7 β β EV6, Location: Nayarit, Mexico, Scale: 1.5. A version of this same map zoomed out to show the whole world is available in Additional file 2. Circles with grey outlines represent the positions of whole genotypes, rather than individual SSRs. Size still indicates the scope of the region represented, though genotype markers are strictly larger than SSR markers to allow both to be visible simultaneously at the same coordinate location

RepeatAnalyzer can also produce publication-quality maps of genotype and/or repeat occurrences over a geographic area. These maps can show any subset of repeats or genotypes and can be filtered by region. If a region filter is applied, the map includes only results that occur in the given region but shows where else they are found in the world. A map can be zoomed to any region of interest, and high-resolution versions can be saved at any zoom level. Figures 4 and 5 show maps resulting from two example queries. The scale of markers on the map can be set from 50 % of the default size up to 300 % of the default size as needed. Figure 5 has been zoomed in to show Mexico, and the full world version is available in Additional file 2.

### Preliminary analysis with *S. pneumoniae* data

To illustrate RepeatAnalyzer’s flexibility with respect to subject species, we collected *S. pneumoniae pspA* gene sequences from GenBank and genotyped the strains using RepeatAnalyzer. From the five sequences we downloaded, we identified 21 unique repeat sequences, each containing 20 to 21 amino acids. These SSR sequences are available in Additional file 3. The genes we examined contained 9 to 16 tandem SSRs. We found that each genotype was distinct (Table 5). Given the length and variability of repeats present in the *pspA* gene in virulent strains of *S. pneumoniae,* we suspect PspA SSR genotypes could be used to differentiate strains with a much finer discrimination than serotyping.

**Table 5:** SSR genotypes of *S. pneumoniae pspA*

Accession #	SSR Repeat pattern^a^
FQ312027	1 2 3 4 5 6 3 4 5 6 3 4 5 6 3 7
ABJ54172	1 8 9 10 9 10 9 10 3 7
U89711	1 8 9 9 11 11 12 13 14
ACB89372	1 16 17 18 19 20 9 6 21 7
AAK74303	15 16 3 4 11 11 10 6 3 7

^a^Repeat sequences are presented in Additional file 3

## Discussion

We developed RepeatAnalyzer, a program for tracking, managing, analysing and cataloguing SSRs and genotypes. RepeatAnalyzer automates naming of new SSRs to avoid the most common types of errors found in analyzing this type of data, and provides new metrics for SSR analysis. As mentioned briefly in the Implementation section, the metrics GD2 and GD2b shown in Table 1 are dependent on the length of genotypes in a region. For example, consider these two cases. Case 1: each genotype in a region has no repeating SSRs, say the strains found there are A B C, D E F and H I J. Case 2: a similar region with strains A B C D E F, H I J L M N, and O P Q R S T. In Case 1, the GD2 value is 200, where as in Case 2 it is 600. Intuitively, both cases have the maximum diversity possible, however increasing the length of the genotypes increases the diversity score, making values difficult to compare between areas with varying genotype lengths. GD2 captures a kind of diversity where more SSRs in the study population will raise the diversity score, while more strains with similar genotypes will decrease the score. In contrast, the GDM1 scores are more focused. GDM1-Global is the average percent of unique repeats across all genotypes in the region and GDM1-Local is the average percent of unique repeats in each genotype. For Case 1 in our example, both the GDM1-Global and GDM1-Local scores would be 1, or 100 %, indicating the maximum possible diversity is present. We argue these values are easier to interpret and are more meaningful. The second type of metric, GDM2 measures how often each repeat in a region occurs, and thus captures a completely different formulation of diversity. For Case 2 of our example, the GDM2-Global and GDM2-Local diversity measures are both 0. This tells us that both in each genotype and in general, each repeat occurs the same number of times.

The validation method we chose to use for RepeatAnalyzer, though it allowed us to test our identification functionality in a large set of test cases, relies on the assumption that GenBank data is correct. In particular, if a DNA sequence is reported correctly in a paper and uploaded incorrectly to GenBank, our validation method would mark that as an error. This can be viewed as a limitation. However, due to the rarity of a mismatch between GenBank and the literature we chose to trust the veracity of GenBank data.

When compiling *A. marginale* data, we found that the naming standards for strains vary considerably across sources. We found some papers [33, 45] that opted against naming strains at all, perhaps due to the uncertainty faced on how best to name strains. A naming scheme containing the location of the strain and the genotype was proposed by Cabezas-Cruz [32]. However, while an improvement, this nomenclature system leaves out year, which nonetheless might be an important piece of information in the future, for example, to determine whether the same genotype is occurring year after year in a particular region. Motivated by this, we propose a new naming convention that is short, information-rich and can be produced directly by RepeatAnalyzer: [genotype]_[country code,province code]_[year]_[animal id]. As an example, using this naming convention, a previously unnamed strain from Kansas would be named 6(D)E_US,KS_2004_8416 [48]. Adding animal id allows someone to quickly realize that in the 2004 Kansas study the 6(D)E genotype occurred seven times.

## Conclusion

In summary, we have developed a new software tool for storing, managing, identifying and analysing short-sequence repeats for the purpose of strain identification. Our software can take a gene sequence and return the repeats it contains along with the known strain (if any) that the sequence belongs to. It does so by storing data distilled from sources on repeats at a given SSR locus. The data can be updated simply and searched easily for information about any known strains or repeats. All of these tasks are done in a computationally efficient manner using the KMP string matching algorithm and general programming best practices. RepeatAnalyzer can also produce a map for any combination of repeats and strains in a given region, offering geographic insights into their distribution not previously available. In addition, it can calculate metrics of diversity within geographic regions. We intend to maintain a periodically updated version of the *A. marginale* data that researchers can download and make contributions to.

## Additional files

Additional file 1: A table including all known A. marginale SSRs. Each SSR includes all names it has been assigned, separated by semicolons, and sources where it has been referenced are cited from the main text. (XLS 68.0 kb) Additional file 2:
**Figure S1.** Geographic Visualization of repeats (Zoomed in to Venezuela from Figure 4). The figure shows the output of the query: Repeats: 10; 11; 12; 13; 14; 15; B; C; α; β; Γ, Strains: None, Location: Any, Scale: 1. **Figure S2.** Geographic Visualization of repeats (Zoomed out from Figure 5). The figure shows the output of the query: Repeats: α; β; Γ; EV1; EV3; EV7; EV6, Strains: EV1 β β β Γ; α β β β Γ; EV1 β β Γ; EV3 EV7 β β EV6, Location: Nayarit, Mexico, Scale: 1.5. (PDF 689 kb) Additional file 3: A table including all SSRs extracted from the *S. pneumoniae* samples. Each SSR is identified by a number. (XLS 28 kb)

## References

[1] Van Belkum A, Scherer S, van Alphen L, Verbrugh H (1998). Short-sequence DNA repeats in prokaryotic genomes. Microbiol Mol Biol Rev.

[2] Hood DW, Deadman ME, Jennings MP, Bisercic M, Fleischmann RD, Venter JC, Moxon ER (1996). DNA repeats identify novel virulence genes in *Haemophilus influenzae*. Proc Natl Acad Sci U S A.

[3] Goh S, Byrne SK, Zhang JL, Chow AW (1992). Molecular typing of *Staphylococcus aureus* on the basis of coagulase gene polymorphisms. J Clin Microbiol.

[4] Hollingshead SK, Becker R, Briles DE (2000). Diversity of PspA: mosaic genes and evidence for past recombination in *Streptococcus pneumoniae*. Infect Immun.

[5] Yother J, Briles DE (1992). Structural properties and evolutionary relationships of PspA, a surface protein of *Streptococcus pneumoniae*, as revealed by sequence analysis. J Bacteriol.

[6] Allred DR, McGuire TC, Palmer GH, Leib SR, Harkins TM, McElwain TF, Barbet AF (1990). Molecular basis for surface antigen size polymorphisms and conservation of a neutralization-sensitive epitope in Anaplasma marginale. Proc Natl Acad Sci.

[7] Benson G (1999). Tandem repeats finder: a program to analyze DNA sequences. Nucleic Acids Res.

[8] Dsouza M, Larsen N, Overbeek R (1997). Searching for patterns in genomic data. Trends Genet.

[9] Farre D (2003). Identification of patterns in biological sequences at the ALGGEN server: PROMO and MALGEN. Nucleic Acids Res.

[10] Camacho C, Coulouris G, Avagyan V, Ma N, Papadopoulos J, Bealer K, Madden TL (2009). BLAST+: architecture and applications. BMC Bioinformatics.

[11] Bao W, Kojima KK, Kohany O (2015). Repbase Update, a database of repetitive elements in eukaryotic genomes. Mob DNA.

[12] Kohany O, Gentles AJ, Hankus L, Jurka J (2006). Annotation, submission and screening of repetitive elements in Repbase: RepbaseSubmitter and Censor. BMC Bioinformatics.

[13] Smit A, Hubley R, Green P. RepeatMasker Open-4.0 2013. http://repeatmasker.org. Accessed 8 Oct 2015.

[14] Kumar P, Chaitanya PS, Nagarajaram H a (2011). PSSRdb: a relational database of polymorphic simple sequence repeats extracted from prokaryotic genomes. Nucleic Acids Res.

[15] Sharma D, Issac B, Raghava GPS, Ramaswamy R (2004). Spectral Repeat Finder (SRF): identification of repetitive sequences using Fourier transformation. Bioinformatics.

[16] Kurtz S, Choudhuri JV, Ohlebusch E, Schleiermacher C, Stoye J, Giegerich R (2001). REPuter: the manifold applications of repeat analysis on a genomic scale. Nucleic Acids Res.

[17] Betley JN, Frith MC, Graber JH, Choo S, Deshler JO (2002). A ubiquitous and conserved signal for RNA localization in chordates. Curr Biol.

[18] Rice P, Longden I, Bleasby A (2000). EMBOSS: the European molecular biology open software suite. Trends Genet.

[19] Bao Z, Eddy SR (2002). Automated de novo identification of repeat sequence families in sequenced genomes. Genome Res.

[20] Grissa I, Vergnaud G, Pourcel C (2007). CRISPRFinder: a web tool to identify clustered regularly interspaced short palindromic repeats. Nucleic Acids Res.

[21] Volfovsky N, Haas BJ, Salzberg SL (2001). A clustering method for repeat analysis in DNA sequences. Genome Biol.

[22] Pellegrini M, Marcotte EM, Yeates TO (1999). A fast algorithm for genome-wide analysis of proteins with repeated sequences. Proteins.

[23] George R a, Heringa J (2000). The REPRO server: finding protein internal sequence repeats through the Web. Trends Biochem Sci.

[24] Edgar RC, Myers EW (2005). PILER: Identification and classification of genomic repeats. Bioinformatics.

[25] Sharma PC, Grover A, Kahl G (2007). Mining microsatellites in eukaryotic genomes. Trends Biotechnol.

[26] Merkel A, Gemmell N (2008). Detecting short tandem repeats from genome data: Opening the software black box. Brief Bioinform.

[27] Lim KG, Kwoh CK, Hsu LY, Wirawan A (2013). Review of tandem repeat search tools: a systematic approach to evaluating algorithmic performance. Brief Bioinform.

[28] Hoffman C. Match patterns in *A. marginale* http://greenbarnstar.github.io/anaplasma-coat. Accessed 2 Nov 2015.

[29] Boyer RS, Moore JS (1977). A fast string searching algorithm. Commun ACM.

[30] Farach M. Optimal Suffix Tree Construction with Large Alphabets. *FOCS’97 Proc 38th Annu Symp Found Comput Sci* 1997:137–143.

[31] Mtshali MS, De La Fuente J, Ruybal P, Kocan KM, Vicente J, Mbati PA, Shkap V, Blouin EF, Mohale NE, Moloi TP, Spickett AM, Latif AA (2007). Prevalence and genetic diversity of *Anaplasma marginale* strains in cattle in South Africa. Zoonoses Public Health.

[32] Cabezas-Cruz A, Passos LMF, Lis K, Kenneil R, Valdés JJ, Ferrolho J, Tonk M, Pohl AE, Grubhoffer L, Zweygarth E, Shkap V, Ribeiro MFB, Estrada-Peña A, Kocan KM, de la Fuente J (2013). Functional and immunological relevance of *Anaplasma marginale* major surface protein 1a sequence and structural analysis. PLoS One.

[33] Castañeda-Ortiz EJ, Ueti MW, Camacho-Nuez M, Mosqueda JJ, Mousel MR, Johnson WC, Palmer GH (2015). Association of *Anaplasma marginale* strain superinfection with infection prevalence within tropical regions. PLoS One.

[34] De la Fuente J, Lew A, Lutz H, Meli ML, Hofmann-Lehmann R, Shkap V, Molad T, Mangold AJ, Almazán C, Naranjo V, Gortázar C, Torina A, Caracappa S, García-Pérez AL, Barral M, Oporto B, Ceci L, Carelli G, Blouin EF, Kocan KM (2005). Genetic diversity of *Anaplasma* species major surface proteins and implications for anaplasmosis serodiagnosis and vaccine development. Anim Health Res Rev.

[35] De La Fuente J, Passos LMF, Van Den Bussche R a, Ribeiro MFB, Facury-Filho EJ, Kocan KM (2004). Genetic diversity and molecular phylogeny of *Anaplasma marginale* isolates from Minas Gerais, Brazil. Vet Parasitol.

[36] De la Fuente J, Ruybal P, Mtshali MS, Naranjo V, Shuqing L, Mangold AJ, Rodríguez SD, Jiménez R, Vicente J, Moretta R, Torina A, Almazán C, Mbati PM, de Echaide ST, Farber M, Rosario-Cruz R, Gortazar C, Kocan KM (2007). Analysis of world strains of *Anaplasma marginale* using major surface protein 1a repeat sequences. Vet Microbiol.

[37] De La Fuente J, Torina A, Caracappa S, Tumino G, Furlá R, Almazán C, Kocan KM (2005). Serologic and molecular characterization of *Anaplasma* species infection in farm animals and ticks from Sicily. Vet Parasitol.

[38] Lew AE, Bock RE, Minchin CM, Masaka S (2002). A msp1α polymerase chain reaction assay for specific detection and differentiation of *Anaplasma marginale* isolates. Vet Microbiol.

[39] Molad T, Fleidrovich L, Mazuz M, Fish L, Leibovitz B, Krigel Y, Shkap V (2009). Genetic diversity of major surface protein 1a of *Anaplasma marginale* in beef cattle. Vet Microbiol.

[40] Mutshembele AM, Cabezas-Cruz A, Mtshali MS, Thekisoe OMM, Galindo RC, de la Fuente J (2014). Epidemiology and evolution of the genetic variability of *Anaplasma marginale* in South Africa. Ticks Tick Borne Dis.

[41] Palmer GH, Rurangirwa FR, Mcelwain TF (2001). Strain composition of the Ehrlichia *Anaplasma marginale* within persistently infected cattle, a mammalian reservoir for tick transmission. J Clin Microbiol.

[42] Pohl AE, Cabezas-Cruz A, Ribeiro MFB, Da Silveira JAG, Silaghi C, Pfister K, Passos LMF (2013). Detection of genetic diversity of *Anaplasma marginale* isolates in Minas Gerais, Brazil. Rev Bras Parasitol Vet.

[43] Ruybal P, Moretta R, Perez A, Petrigh R, Zimmer P, Alcaraz E, Echaide I, Torioni de Echaide S, Kocan KM, de la Fuente J, Farber M (2009). Genetic diversity of *Anaplasma marginale* in Argentina. Vet Parasitol.

[44] Silva JB, Fonseca AH, Barbosa JD, Cabezas-Cruz A, de la Fuente J (2014). Low genetic diversity associated with low prevalence of *Anaplasma marginale* in water buffaloes in Marajó Island, Brazil. Ticks Tick Borne Dis.

[45] Ybañez AP, Ybañez RHD, Claveria FG, Cruz-Flores MJ, Xuenan X, Yokoyama N, Inokuma H (2014). High genetic diversity of *Anaplasma marginale* detected from Philippine Cattle. J Vet Med Sci.

[46] Rodríguez J-L, Palmer GH, Knowles DP, Brayton KA (2005). Distinctly different msp2 pseudogene repertoires in *Anaplasma marginale* strains that are capable of superinfection. Gene.

[47] Ooshiro M, Zakimi S, Matsukawa Y, Yafuso M, Katagiri Y, Inokuma H (2009). *Anaplasma marginale* infection in a Japanese Black cow 13 years after eradication of *Rhipicephalus (Boophilus) microplus* in Okinawa, Japan. Vet Parasitol.

[48] Palmer GH, Knowles DP, Rodriguez J, Gnad DP, Hollis LC, Marston T, Brayton KA (2004). Stochastic Transmission of Multiple Genotypically Distinct *Anaplasma marginale* Strains in a Herd with High Prevalence of Anaplasma Infection. J Clin Microbiol.

[49] Futse JE, Ueti MW, Knowles DP, Palmer GH (2003). Transmission of *Anaplasma marginale* by *Boophilus microplus*: retention of vector competence in the absence of vector-pathogen interaction. J Clin Microbiol.

[50] De la Fuente J, Thomas EJG, Den Bussche V, Ronald A, Hamilton RG, Tanaka EE, Druhan SE, Kocan KM (2003). Characterization of Anaplasma marginale Isolated from North American Bison characterization of *Anaplasma marginale* isolated from North American Bison. Appl Environ Microbiol.

[51] Benson D a, Karsch-Mizrachi I, Lipman DJ, Ostell J, Wheeler DL (2007). GenBank. Nucleic Acids Res.

