# Supplementary material for: RepeatAnalyzer: a tool for analysing and managing short-sequence repeat data
Source: BMC Genomics. 2016 Jun 3;17:422. doi: 10.1186/s12864-016-2686-2 (PMC4891823; doi:10.1186/s12864-016-2686-2)
Supplement: Additional file 2: — Figure S1. Geographic Visualization of repeats (Zoomed in to Venezuela from Figure 4). The figure shows the output of the query: Repeats: 10; 11; 12; 13; 14; 15; B; C; α; β; Γ, Strains: None, Location: Any, Scale: 1. Figure S2. Geographic Visualization of repeats (Zoomed out from Figure 5). The figure shows the output of the query: Repeats: α; β; Γ; EV1; EV3; EV7; EV6, Strains: EV1 β β β Γ; α β β β Γ; EV1 β β Γ; EV3 EV7 β β EV6, Location: Nayarit, Mexico, Scale: 1.5. (PDF 689 kb) [file 12864_2016_2686_MOESM2_ESM.pdf]

Supplementary Figure 1. Geographic Visualization of repeats (Zoomed in to Venezuela from Figure 4). The figure shows the output of the query: Repeats: 10; 11; 12; 13; 14; 15; B; C; α; β; Γ, Strains: None, Location: Any, Scale: 1.

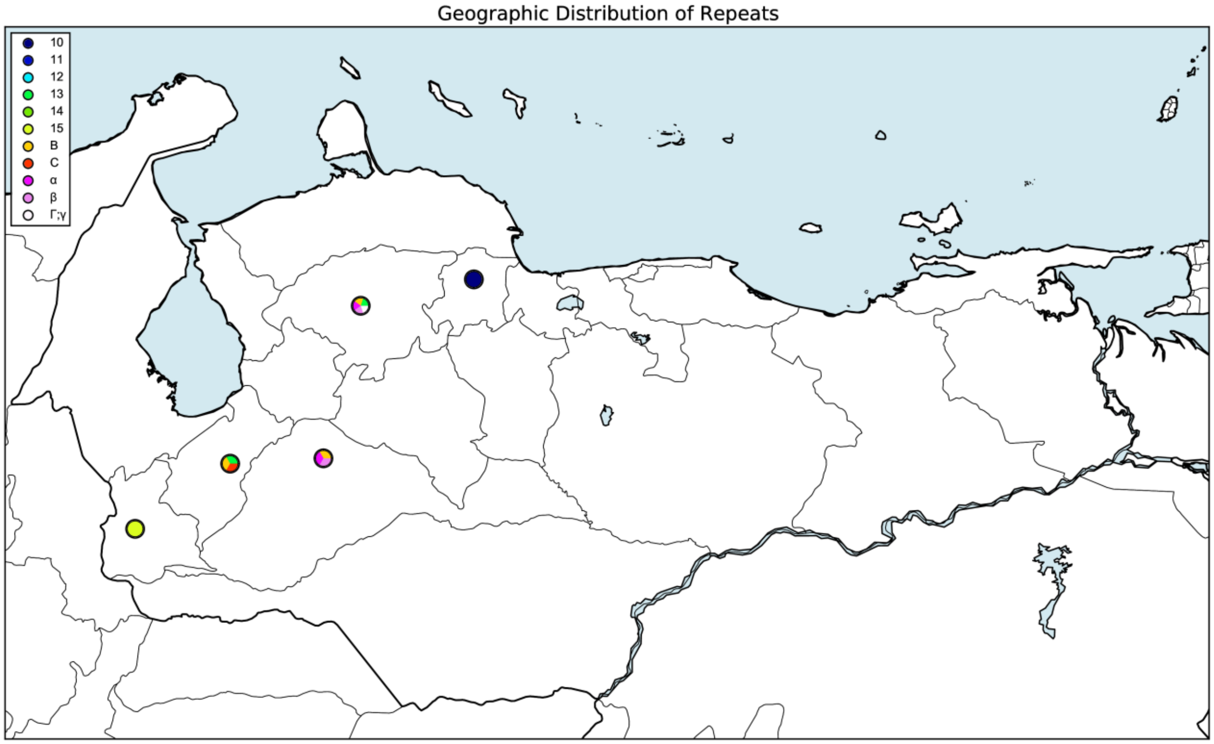

Supplementary Figure 2. Geographic Visualization of repeats (Zoomed out from Figure 5). The figure shows the output of the query: Repeats: α; β; Γ; EV1; EV3; EV7; EV6, Strains: EV1 β β β Γ; α β β β Γ; EV1 β β Γ; EV3 EV7 β β EV6, Location: Nayarit, Mexico, Scale: 1.5.

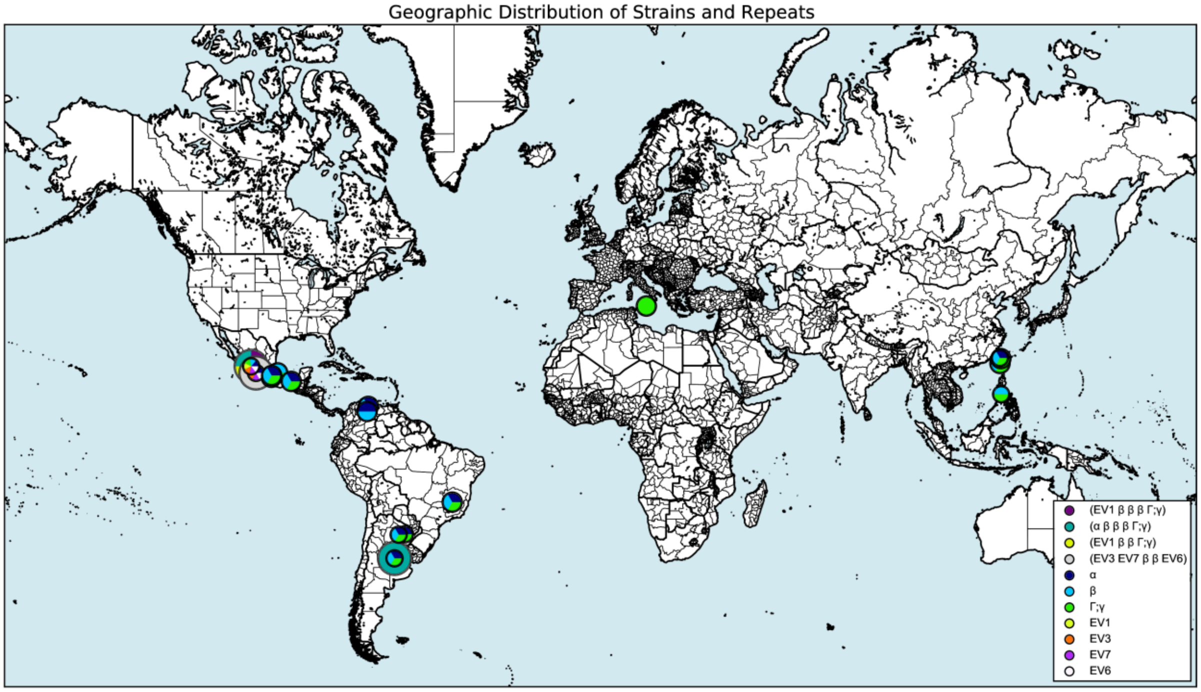

Dark grey borders correspond to genotypes while black borders correspond to repeats. Larger circles correspond to larger scope areas. For instance, a circle representing strains in the U. S. A. would be larger than one corresponding to strains in Texas.
